# Epithelial Antimicrobial Peptide/Protein and Cytokine Expression Profiles Obtained from Nasopharyngeal Swabs of SARS-CoV-2-Infected and Non-Infected Subjects

**DOI:** 10.3390/v16091471

**Published:** 2024-09-15

**Authors:** Thilo Gambichler, Silke Goesmann, Marina Skrygan, Laura Susok, Christian Schütte, Nahza Hamdani, Wolfgang Schmidt

**Affiliations:** 1 Department of Dermatology, Ruhr-University Bochum, 44791 Bochum, Germany; silke.goesmann@rub.de (S.G.); marina.skrygan@klinikum-bochum.de (M.S.); laura.susok@klinikumdo.de (L.S.); 2 Department of Dermatology, Dortmund Hospital, Faculty of Health, School of Medicine, University Witten/Herdecke, 44137 Dortmund, Germany; 3 Department of Dermatology, Christian Hospital Unna, 59423 Unna, Germany; 4 Department of Internal Medicine, Ruhr-University Bochum, 44791 Bochum, Germany; christian.schuette@rub.de (C.S.); w.schmidt@rub.de (W.S.); 5 Department of Molecular and Experimental Cardiology, Ruhr-University Bochum, 44791 Bochum, Germany; nazha.hamdani@rub.de; 6 Department of Cardiology, Ruhr-University Bochum, 44791 Bochum, Germany; 7 Institute of Physiology, Ruhr-University Bochum, 44801 Bochum, Germany

**Keywords:** SARS-CoV-2, COVID-19, antimicrobial peptides, antimicrobial proteins, innate immunity, pro-inflammatory cytokines, coronavirus, S100A7, psoriasin, SALP/elafin, RNase-7

## Abstract

Immune responses of the epithelia of the upper respiratory tract are likely crucial in early inhibition of the viral replication and finally clearance of SARS-CoV-2. We aimed to compare the expression profiles of antimicrobial peptides/proteins (AMPs) and related cytokines observed in the nasopharynx of SARS-CoV-2-infected patients and non-infected controls and to assess the associations between these parameters and COVID-19 patients’ outcomes. We included 45 subjects who had tested positive for SARS-CoV-2 and 22 control subjects who had tested negative for SARS-CoV-2. Biomaterial for SARS-CoV-2 detection, as well as gene and protein expression studies, was obtained from all subjects using nasopharyngeal swabs which were performed a maximum of 7 days before inclusion in the study. Univariable and multivariable statistics were performed. When compared to the controls, the mRNA expression levels of human β-defensin 1 (hBD-1), LL-37, and trappin-2 were significantly higher in specimens of nasopharyngeal swabs from COVID-19 patients. Protein expression of hBD-1 was also increased in the COVID-19 group. mRNA expression levels of interferon-ɣ (IFN-ɣ), tumor necrosis factor- ɑ (TNF-ɑ), and interleukin-6 (IL-6) measured in SARS-CoV-2-infected patients were significantly higher than those observed in the controls, which could also be confirmed in the protein levels of IFN-ɣ and IL-6. A significant correlation between mRNA and protein levels could be observed only for IL-6. Univariable analysis revealed that low IFN-ɣ mRNA levels were associated with severe/fatal outcomes. The occurrence of COVID-19 pneumonia was significantly associated with lower expression levels of IL-6 mRNA, IFN-ɣ mRNA, and TNF-ɑ mRNA. Concerning the severe/fatal outcomes, the multivariable logistic regression model revealed that none of the aforementioned parameters remained significant in the model. However, the logistic regression model revealed that higher TNF-ɑ mRNA expression was a significant independent predictor of absence of pneumonia [odds ratio: 0.35 (95% CI 0.14 to 0.88, *p* = 0.024)]. In conclusion, nasopharyngeal expression of AMPs (hBD-1, LL-37, and trappin-2) and cytokines (IL-6, IFN-ɣ, and TNF-ɑ) is upregulated in response to early SARS-CoV-2 infection, indicating that these AMPs and cytokines play a role in the local host defense against the virus. Upregulated nasopharyngeal TNF-ɑ mRNA expression during the early phase of SARS-CoV-2 infection was a significant independent predictor of the absence of COVID-19 pneumonia. Hence, high TNF-ɑ mRNA expression in the nasopharynx appears to be a protective factor for lung complications in COVID-19 patients.

## 1. Introduction

The emergence of the SARS-CoV-2 pandemic in December 2019 has resulted in an unprecedented global social and economic impact on humanity. Infection with SARS-CoV-2 is the cause of the coronavirus disease 2019 (COVID-19), a condition that has, unfortunately, claimed approximately 7 million lives worldwide [1]. The risk of severe and fatal COVID-19 is frequently associated with an advanced age, cardiovascular conditions, diabetes, obesity, chronic lung diseases, chronic liver conditions—including metabolic dysfunction-associated steatotic liver disease—hypertension, and malignancies. Laboratory predictors of severe and fatal COVID-19 predominantly include eosinopenia, elevated lactate dehydrogenase, C-reactive protein, procalcitonin, the neutrophil-to-lymphocyte ratio, and D-dimers. Additionally, increased serum levels of cytokines, such as interleukin-6 (IL-6), IL-8, IL-10, tumor necrosis factor-alpha (TNF-ɑ), and interferons, as well as decreased lymphocyte counts, particularly CD8+ lymphocytes and natural killer cells, have been observed [2,3,4,5,6].

Although most clinical investigations have focused on humoral and cellular immunity in the peripheral blood, immune responses in the epithelial tissues of the upper respiratory tract are likely crucial for early inhibition of viral replication and the eventual clearance of SARS-CoV-2 [7]. Therefore, an innate immune response plays a key role in managing SARS-CoV-2 replication and controlling symptoms in the early stages of COVID-19 [8]. The antiviral innate immune response, including the complement system, interferons (IFNs), chemokines, immune cells (e.g., macrophages), and antimicrobial peptides/proteins (AMPs), plays an important role during the initial phases of SARS-CoV-2 infection. These components primarily function by limiting viral spread through cytokine modulation and by inducing adaptive immune responses. A failure of the innate immune response against SARS-CoV-2 can lead to aberrant acquired immune responses, potentially resulting in a critical course of the disease [9,10,11,12,13,14,15,16,17,18,19,20,21,22,23,24,25,26,27,28,29,30,31,32,33,34,35,36,37,38,39,40].

Defensins (α and β) are AMPs that act against various bacterial, fungal, and viral pathogens. Human β-defensins (hBDs) 1–4 have overlapping expression patterns, with hBD-1 being produced and expressed in the respiratory tract epithelia, which are in direct contact with ambient microflora. hBDs play a crucial role in inflammatory processes by stimulating antigen-presenting cells, such as dendritic cells, leading to T lymphocyte activation and the initiation of adaptive immune responses. In addition to its antimicrobial activity, human β-defensin-2 (hBD-2) can promote the production of pro-inflammatory mediators, including chemokines and cytokines, such as IL-6 and IL-10, to combat infections [8,12,27,33,34,35,39,41]. Another AMP, LL-37, from the cathelicidin family, exerts strong antiviral, antibacterial, and immunomodulatory effects by stimulating the production of cytokines (e.g., IL-6) and chemokines (e.g., CXCL10), promoting leukocyte chemotaxis, and enhancing the differentiation of innate immune cells such as macrophages and dendritic cells [12,32,33,36,38,39]. However, epithelial cells of the upper airway system also express other AMPs, including S100A7 (psoriasin), RNase-7, and trappin-2 (SKALP/elafin), which have yet to be studied in the context of SARS-CoV-2 infection. In the present study, we aimed to compare the expression profiles of a broader panel of AMPs and related cytokines observed in the nasopharynx of SARS-CoV-2-infected patients and non-infected controls, and to assess the associations between these parameters and COVID-19 patient outcomes [12,13,14,15].

## 2. Materials and Methods

### 2.1. Patients and Controls

The present prospective study (ethical approval: #21-7284, 08.06.2021) included 45 subjects who had tested positive for SARS-CoV-2 and 22 control subjects who had tested negative. Biomaterial for SARS-CoV-2 detection, as well as gene and protein expression studies, was obtained from all subjects using nasopharyngeal swabs which were performed a maximum of 7 days before inclusion in the study [median (range) days: 2 (1–7)]. All nasal swabs with a Ct-value below 40 were included in the study. The control subjects, who had a negative SARS-CoV-2 test, had no history of previous COVID-19 infection, contact with infected patients, or any COVID-19 characteristic symptoms within the last week before inclusion in the study. We also made sure to match the SARS-CoV-2-infected subjects and controls concerning age, gender, immunosuppression, and relevant comorbidities, as much as possible. Selected subjects did not have any other known infections at the time of diagnosis, except for COVID-19. The biomaterial collected for the expression studies was centrifuged with 1000 g at 2–8 °C for 20 min. The cell pellet was used for gene expression analysis using a qRT-PCR assay. The supernatant was examined utilizing ELISA for a protein expression analysis. The WHO clinical progression scale was used for COVID-19 severity classification, providing a measure of illness severity across a range from 0 (not infected) to 10 (death), and grouping these in stages: I = score 1–3; II = score 4 and 5; III = score 6–9; and IV = score 10 [13]. For statistical analysis, we dichotomized the WHO clinical progression scale by grouping stages I and II vs. III and IV [42].

### 2.2. qRT-PCR

SARS-CoV-2 was detected using a commercial qRT-PCR assay (AllplexTM 2019-nCoV, Seegene, Seoul, Republic of Korea) according to a standard protocol. Thus, the infection status, including Ct-values, was determined using this assay. Using the lysed cell pellets from qRT-PCR for antimicrobial gene expression, an analysis was also performed on SARS-CoV-2-infected and non-infected subjects for AMPs (hBD-1, hBD-2, S100A7, LL-37, RNase-7, and trappin-2) and pro-inflammatory cytokines (IL-6, TNF-ɑ, and IFN-ɣ). The primers used for qRT-PCR were developed in our internal laboratory using the Primer Express software version 3.0. The primers were specifically designed for mRNA. The qPCR was carried out with Power SYBR Green PCR Mastermix (Thermo Fisher, Waltham, MA, USA) using QuantStudio 5 (Applied Biosystems, Waltham, MA, USA).

### 2.3. ELISA

The cell supernatant from the nasopharyngeal swab was analyzed using the ELISA technique. ELISA was only performed for AMPs that significantly differed between COVID-19 patients and controls on qRT-PCR analysis. hBD-1, LL-37, and trappin-2, as well as the pro-inflammatory cytokines IL-6, TNF-α, and IFN-ɣ, were measured and analyzed. Various commercially available ELISA kits were used, including Sandwich ELISA kits for hBD-1 (ABIN6955311) and SKALP (ABIN6958441) from antibodies-online (Germany); LL-37 (HK321) from HycultBiotech (Beutelsbach, Germany); and IL-6 (BMS213-2HS), TNF-ɑ (BMS223-2HS), and IFN-ɣ (BMS228-2HS) from Invitrogen (Schwerte, Germany). All test kits were highly sensitive and specific for the relevant human AMPs and cytokines assessed. The test procedures were performed according to the manufacturer’s instructions, and the concentration of the AMPs and cytokines was determined based on standard curves. The manufacturers specified the assay detection ranges as follows: hBD-1 (0.312–20 ng/mL), LL-37 (0.14–100 ng/mL), trappin-2 (0.31–20 ng/mL), IL-6 (0.08–5 pg/mL), TNF-α (0.31–20 pg/mL), and IFN-ɣ (0.16–10).

### 2.4. Statistical Analysis

The MedCalc (Ostend, Belgium) software version 20.021 was used. Analysis of data distribution was performed by the D’Agostino–Pearson test. Univariable statistics included the Chi^2^ test for dichotomized data and receiver operating characteristics [ROCs, including associated criterion, area under the curve (AUC), and Youden index (optimal cut-off points of both the maximum sensitivity and specificity)], analyses, the variance ratio test (F-test), the Mann–Whitney test, and the Spearman correlation procedure for continuous data. Multivariable analysis was performed using a logistic regression model, exclusively including data obtained from univariable testing if (1) significant with an AUC of ≥0.65 on ROC analysis or (2) significant on Chi^2^ analysis using categorial data. Odds ratios (ORs), including 95% confidence intervals (CIs), were calculated as well. A result of *p* < 0.05 was considered statistically significant.

## 3. Results

### 3.1. Subjects’ Characteristics

Clinical characteristics of patients and controls are detailed in Table 1. Together, we studied 45 patients with COVID-19 infection and 22 non-infected controls. Almost all SARS-CoV-2-infected individuals were inpatients. There was no significant (*p* > 0.1) difference between SARS-CoV-2-infected individuals and non-infected subjects regarding sex, age, relevant comorbidities, and immunosuppression.

The median (range) Ct-value of the COVID-19 patients was 20 (10.1–34.9). During the course of the disease, antiviral therapy was administered in 23 (51.1%) patients (Table 1). We detected the Delta variant in 7 (15.6%) patients and the Omicron variant in 14 (31.1%) patients. For 24 (53.3%) patients, a determination of the virus variant was not performed. Of 45 COVID-19 patients, 35 (77.8%) had received at least one previous SARS-CoV-2 vaccination. Pneumonia was clinically and radiologically diagnosed in 16 (35.6%) patients. A severe to fatal outcome (WHO III/IV) was observed in eight (17.8%) patients, including two (4.4%) COVID-19 deaths. Moreover, risk factors (e.g., diabetes mellitus, cardiovascular conditions, active malignancies, and immunosuppression) were not significantly associated with severe/fatal COVID-19 outcomes, except for a history of lung diseases (*p* = 0.0063) and an age above 62 years (AUC 0.70, *p* = 0.023, Youden index 0.42). The occurrence of COVID-19 pneumonia was associated with pre-existing lung diseases (*p* = 0.011), a more advanced age (*p* = 0.033), and pre-existing cardiovascular diseases (*p* = 0.024).

### 3.2. AMPs and Cytokines in COVID-19 Patients vs. Controls

When compared to the controls, the mRNA expression levels of hBD-1 (*p* = 0.0004), LL-37 (*p* = 0.0027), and trappin-2 (*p* = 0.0024) were significantly higher in the specimens of nasopharyngeal swabs of COVID-19 patients (Table 2). On the protein level, this could only be confirmed for hBD-1 (*p* = 0.0002). mRNA expression levels of IFN-ɣ (*p* = 0.034), TNF-ɑ (*p* < 0.0001), and IL-6 (*p* = 0.0024) measured in SARS-CoV-2-infected patients were significantly higher than those observed in the controls. In the case of IFN-ɣ (*p* = 0.036) and IL-6 (*p* = 0.0093), the mRNA findings could also be confirmed on the protein level (Table 1). A significant correlation between the mRNA and protein expression could only be observed for IL-6 (*r* = 0.55, *p* = 0.010). A positive correlation was found for most combinations of AMPs and cytokines studied, with coefficients of rank correlation ranging from 0.31 to 0.52 (*p* < 0.05).

### 3.3. Association between Clinical Characteristics and AMP and Cytokine Expression Levels

**Figure 1:**
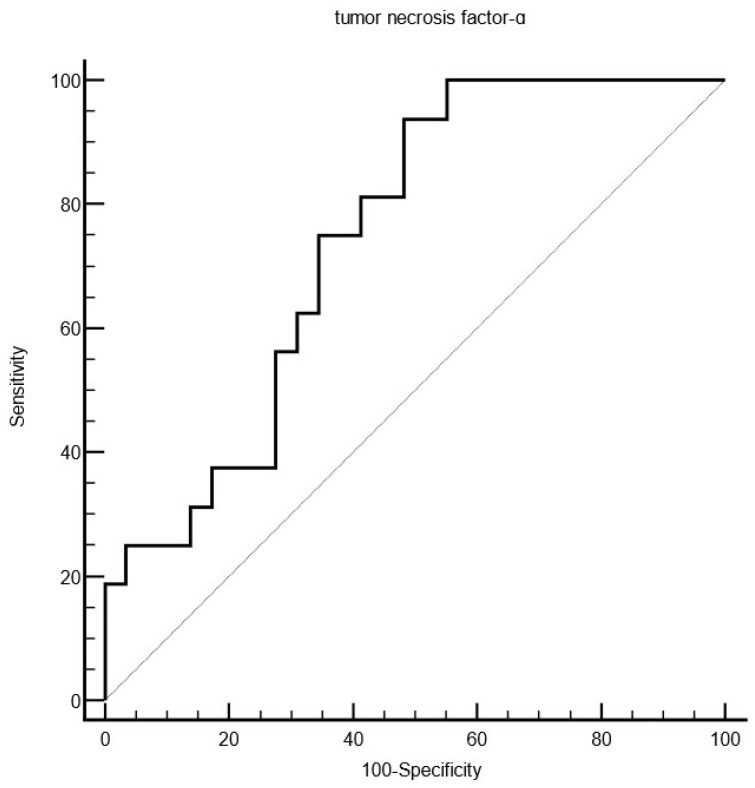
Showing an ROC analysis indicating that lower tumor necrosis factor-α mRNA levels (criterion: ≤1.64, AUC 0.75, *p* = 0.0008, Youden index 0.45) obtained by nasopharyngeal swabs are associated with the occurrence of COVID-19 pneumonia.

In most instances, the AMP expression of COVID-19 patients did not correlate with clinical features (e.g., age, sex, and course of infection), except for S100A7 mRNA expression (*r* = 0.33, *p* = 0.033) and LL-37 mRNA expression (*r* = 0.32, *p* = 0.032), which positively correlated with Ct-values at baseline levels. Univariable analysis revealed that low IFN-ɣ mRNA levels were associated with severe/fatal outcomes (criterion: ≤0.11, AUC 0.73, *p* = 0.0095, Youden index 0.58). The occurrence of COVID-19 pneumonia was significantly associated with lower expression levels of IL-6 mRNA (criterion: ≤0.28, AUC 0.74, *p* = 0.0043, Youden index 0.47), IFN-ɣ mRNA (criterion: 0.15, AUC 0.83, *p* > 0.0001, Youden index 0.65), and TNF-α mRNA (criterion: ≤1.64, AUC 0.75, *p* = 0.0008, Youden index 0.45, Figure 1).

Using all significant variables obtained from univariable analysis (IFN-ɣ mRNA, pre-existing lung disease, and an age above 62 years), the multivariable logistic regression model for disease severity (WHO III/IV) revealed that none of the aforementioned parameters remained significant in the model. With regards to the absence of pneumonia, however, the logistic regression model revealed that TNF-ɑ mRNA detected in nasopharyngeal swab specimens of COVID-19 patients was the only significant independent predictor, as indicated by an odds ratio of 0.35 (95% CI 0.14 to 0.88, *p* = 0.024). The other parameters (pre-existing lung and cardiovascular diseases, older age, IL-6 mRNA, and IFN-ɣ mRNA) did not remain significant in the logistic regression model.

## 4. Discussion

In the present study, we demonstrated that mRNA expression levels of hBD-1, LL-37, and trappin-2 were significantly higher in nasopharyngeal swab specimens from COVID-19 patients compared to non-infected controls. Additionally, hBD-1 protein expression was also increased in COVID-19 patients compared to the controls. However, no significant differences were observed for hBD-2 and RNase-7. Moreover, the AMPs assessed in this study were not significantly associated with clinical parameters or patient outcomes, except for S100A7 and LL-37, which showed a positive correlation with Ct-values in COVID-19 patients.

Previous studies primarily focused on hBD expression in the serum of COVID-19 patients, which cannot be directly compared with the expression profiles in the nasopharynx. For example, Al-Bayatee and Ad’hiah [43] reported low serum levels of hBD-2 in patients with severe COVID-19, while hBD-4 levels were elevated. In line with our findings, Brancaccio et al. [44] demonstrated that pregnant women with COVID-19 showed an increase in pro-inflammatory cytokines (e.g., TNF-α, IL-6, and IL-8) and AMPs (e.g., hBD-1, hBD-2, and hBD-4) in their serum, indicating an enhanced immune response against SARS-CoV-2 infection.

More data are available on the significance of LL-37 in combating SARS-CoV-2 infection. Recently, Aloul et al. [45] reviewed the relevance of human cathelicidin LL-37 in COVID-19, highlighting that its effects extend beyond inhibiting SARS-CoV-2 replication and infection. Keutmann et al. [36] indicated that the serum LL-37-to-leukocyte count ratio could be used to assess the risk of COVID-19 progression at the time of hospital admission. De Buhr et al. [46] identified two potential risk factors in elderly male patients—reduced DNase activity and increased LL-37 plasma levels—that may lead to an inefficient neutrophil extracellular trap (NET) degradation and a higher risk of NET-associated thrombosis during COVID-19.

By contrast, recent studies suggest the potential therapeutic and prophylactic uses of LL-37 as an effective tool in mitigating COVID-19 pathology and reducing severe effects of infection [38,39,47,48]. Eisenhut and Shin [47] demonstrated that the primary prevention of coronavirus infection and the suppression of TNF-α release could be enhanced by the induction of AMPs such as LL-37 and hBD-2. Notably, Roth et al. [48] and Li et al. [38] showed that LL-37 can inhibit the binding of the SARS-CoV-2 spike protein to its cellular receptor, angiotensin-converting enzyme 2 (ACE2).

Interestingly, Ferrucci et al. [39] recently reported that prophylactic treatment with a nutraceutical formula (Solution-3) resulted in immunomodulation in epithelial cells, including those in the upper respiratory tract. This formula negatively modulated NF-κB signaling by decreasing the phosphorylation of the NF-κB protein p65, enhanced the expression of inflammatory cytokines (e.g., IFN-γ, TNF-α, and IL-6), affected the expression of IL-1β and CXCL10, and induced the secretion of hBD-2 and LL-37. These effects could lead to the activation of innate immune processes, including dendritic cell maturation, macrophage and neutrophil chemotaxis, and CD4+ T cell activation. Notably, Solution-3 (e.g., nano spray) appears to be an interesting immunostimulant that may be beneficial as a preventive measure or even to combat early-stage SARS-CoV-2 infection [39].

Trappin-2 (SKALP/elafin), which is a potent inhibitor of neutrophil serine proteases, has not been studied in COVID-19 patients. However, data from Tanga et al. [15] indicate that an engineered trappin-2 A62L variant is a powerful anti-protease and anti-inflammatory agent that could potentially be developed to treat patients with inflammatory lung diseases. Our study is the first to show that trappin-2 mRNA expression is upregulated in the epithelial cells of the nasopharynx in COVID-19 patients, suggesting that trappin-2 plays a role in the initial innate immune response to SARS-CoV-2 infection in the upper airway tract.

In addition to the upregulation of AMPs in the nasopharynx during SARS-CoV-2 infection, we observed increased gene expression profiles of IFN-γ, TNF-ɑ, and IL-6 in COVID-19 patients, which was further confirmed at the protein level for IFN-γ and IL-6. Moreover, we found positive correlations between most combinations of AMPs and cytokines studied, indicating that AMP and cytokine upregulation occurs in parallel during the initial phase of SARS-CoV-2 infection in the nasopharyngeal epithelium [21,32,34,39]. Fan et al. [23] showed that coordinated IFN-γ production and clonal expansion of SARS-CoV-2-specific T lymphocytes are associated with disease resolution in COVID-19. Additionally, Cheemarla et al. [49], using single-cell analysis of nasopharyngeal epithelial cells from COVID-19 patients, demonstrated that interferon-stimulated gene induction occurred primarily in patients with mild-to-moderate disease, but not in those with severe COVID-19. This finding supports the observation that patients with severe disease are more likely to have genetic or acquired deficiencies in IFN signaling pathways. Cheemarla et al. [49] concluded that epithelial cells are capable of mounting an IFN response to SARS-CoV-2, but the timing and magnitude of this response relative to viral replication may be critical in determining the course of COVID-19 infection. The prognostic significance of an IFN response in SARS-CoV-2 infection has also been reported by other investigators [17,18,24,28,30].

In the present study, we demonstrated that the occurrence of COVID-19 pneumonia, as well as a severe or fatal occurrence of the disease, was significantly associated with lower expression levels of IFN-ɣ mRNA (criteria: ≤0.16 and ≤0.11, respectively), with high Youden indices of 0.65 and 0.58, respectively. Surprisingly, however, IFN-ɣ mRNA did not remain significant in the multivariable analysis. On the other hand, the logistic regression model revealed that the high TNF-ɑ mRNA expression detected in nasopharyngeal swab specimens from COVID-19 patients was the only significant independent predictor of the absence of COVID-19-associated pneumonia, with a robust odds ratio of 0.35 (95% CI 0.14 to 0.88, *p* = 0.024).

Similar to IFN-ɣ and IL-6 mRNA expression, TNF-α mRNA expression was significantly higher in the nasopharynx of COVID-19 patients than in non-infected subjects. This observation is consistent with the findings of Chiu et al. [50], who reported that TNF-ɑ and IL-6 mRNA expression levels were significantly higher in nasopharyngeal specimens of SARS-CoV-2-infected patients compared to the controls. In line with our findings, at least in univariable testing, Ghulam et al. [51] showed that mRNA expression of cytokines such as IL-6, IFN-ɣ, and TNF-ɑ was significantly decreased in patients with severe COVID-19 compared to those with mild disease. However, the multivariable analysis by Ghulam et al. [51] revealed a significant relationship between age, IL-6, and disease severity. Of course, the results of the present study and Ghulam et al. [51] are not directly comparable, as the dependent variables in the regression models differed between the two studies. These findings suggest that pro-inflammatory cytokine expression in the upper airway during the early stages of infection differs from cytokine expression profiles and outcomes observed in the lungs and circulation during later stages, particularly in critical stages such as the cytokine storm [26,27,28,29,52].

What are the clinical implications that may result from studies in this field? If certain AMPs or cytokines are found to be protective, they could become potential therapeutic targets [47,48]. Conversely, if others are associated with excessive inflammation or tissue damage, modulating their activity might offer a way to reduce harmful immune responses. Understanding the AMP and cytokine levels in the nasopharyngeal tract could lead to the discovery of biomarkers that predict the course of the disease or patient outcomes, leading to earlier intervention and more personalized treatment strategies. Insights from these studies could also inform vaccine design and strategies for priming the immune system, particularly at mucosal surfaces like the nasopharynx, where the virus first encounters host defenses.

The limitations of the present study include the relatively small and unbalanced number of patients and control subjects. However, the comparability of both groups in terms of age, sex, obesity, and other comorbidities represents a notable strength. The cross-sectional design used could not yield data on the course of infection over time, which had provided more in-depth information on the nasopharyngeal AMP and cytokine expression during the infection. The present study is largely descriptive, focusing on the expression levels of mRNA and proteins without conducting functional assays to determine the biological effects of nasopharyngeal AMPs and cytokines [53]. Hence, the correlations and associations observed were not supported by mechanistic data. Moreover, other parameters may have been missed in the present study which may potentially influence the outcomes. Nevertheless, this is the first descriptive study to investigate a panel of AMPs and related cytokines in the nasopharynx of COVID-19 patients and healthy controls. Of course, our data will have to be substantiated by larger independent studies also including functional test methods.

## 5. Conclusions

In the present study, we showed that the nasopharyngeal expression of hBD-1, LL-37, and trappin-2 is upregulated in response to early SARS-CoV-2 infection, indicating that these AMPs play a role in the local host defense against the virus. However, we did not observe a significant association between AMP expression and the course of COVID-19. Additionally, we observed a significant upregulation of cytokines IL-6, IFN-ɣ, and TNF-α in response to SARS-CoV-2 infection in the nasopharyngeal epithelium. Notably, we found that elevated TNF-α mRNA expression in the nasopharynx during the early phase of infection was a significant independent predictor of the absence of COVID-19 pneumonia. Thus, high nasopharyngeal TNF-ɑ mRNA expression may serve as a protective factor against lung complications in COVID-19 patients.

## Figures and Tables

**Table 1:** Describing clinical characteristics of COVID-19 patients (n = 45) and healthy controls (n = 22).

Parameters	COVID-19	Healthy Controls	*p*-Value
**Sex** m/f	26/19 (57.8%/42.2%)	11/11 (50%/50%)	=0.98
**Median age (range)**	63.4 (17–87) years	59.5 (19–93) years	=0.38
**Relevant comorbidities** ^#^ no/yes	7/38 (15.6%/84.4%)	6/16 (27.3%/72.7%)	=0.86
**Obesity** no/yes	42/3 (93.3%/6.7%)	20/2 (90.9%/9.1%)	=0.99
**Immunosuppression** no/yes	32/13 (71.1%/28.9%)	17/5 (77.3%/22.7%)	= 0.99
**Previous COVID-19** **vaccination** no/yes	10/35 (22.2%/77.8%)	-	-
**Median Ct-value at diagnosis**	20 (10.1–34.9)	-	-
**Therapy against SARS-CoV-2** ^&^ no/yes	22/23 (48.9%/51.1%)	-	-
**Pneumonia** no/yes	29/16 (64.4%/35.6%)	-	-
**Course of disease** asymptomatic to moderate/severe to fatal ^$^; no/yes (WHO III/IV) [42]	37/8 (82.2%/17.8%)	-	-

^#^ cardiovascular conditions, diabetes mellitus, obesity, lung diseases, etc.; ^&^ casirivimab/imdevimab, tocilizumab, sotrovimab, remdesivir, molnupiravir, corticosteroids; ^$^ 2 deaths.

**Table 2:** Antimicrobial peptide/protein and cytokine mRNA and protein * expression levels of COVID-19 patients (n = 45) and healthy controls (n = 22).

mRNA/Protein Expression	COVID-19 Patients	Healthy Controls	*p*-Value
**hBD1 mRNA** **hBD1 protein (ng/mL)**	6.2 (0.83–89.5) 16.4 (9.7–22.3)	2.6 (0.5–19.9) 13.2 (0.1–15.4)	=0.0004 =0.0002
**hBD2 mRNA**	3.6 (0.1–114.9)	3.1 (0.1–38.4)	=0.087
**LL-37 mRNA** LL-37 protein (ng/mL)	0.43 (0.02–3.3) 2.7 (0.33–29.1)	0.16 (0.05–1.48) 3.6 (0.14–8.22)	=0.0027 =0.79
**S100A7 mRNA**	3.8 (0.05–509.8)	1.2 (0.06–41.8)	=0.10
**RNase-7 mRNA**	4.3 (0.32–469.1)	5.1 (0.15–191.9)	=0.45
**Trappin-2 mRNA** Trappin-2 protein (ng/mL)	13.5 (0.15–99.2) 33.6 (25.7–45.3)	5.7 (2.5–80) 31.3 (21.6–42.8)	=0.024 =0.42
**IFN-ɣ mRNA** IFN-ɣ protein (pg/mL)	0.16 (0.02–9.7) 0.77 (0.03–18.4)	0.085 (0.01–0.45) 0.11 (0.04–0.43)	=0.034 =0.036
**IL-6 mRNA** IL-6 protein (pg/mL)	0.33 (0.03–13.1) 13.2 (0.54–33.5)	0.12 (0.01–0.3) 3.3 (0.24–8.6)	=0.0024 =0.0093
**TNF-α mRNA** TNF-α protein (pg/mL)	1.3 (0.06–55.5) 2.3 (0.16–42.9)	0.27 (0.02–1.4) 2.1 (1.33–2.89)	<0.0001 =0.34

* protein expression was only assessed in parameters which significantly differed on mRNA analysis.

## Data Availability

Derived data supporting the findings of this study are available from the corresponding author T.G. on reasonable request.
